# Actinic Keratosis and Human Papillomaviruses: may their relationship constitute a new approach for actinic keratosis management?

**DOI:** 10.1016/j.tvr.2025.200330

**Published:** 2025-11-08

**Authors:** Maria Antonia De Francesco, Martina Salvi, Roberta Gerami, Maria Alberti, Federico Cesanelli, Irene Scarvaglieri, Giorgio Tiecco, Eugenia Quiros-Roldan

**Affiliations:** a Section of Microbiology, Department of Molecular and Translational Medicine, University of Brescia and ASST Spedali Civili di Brescia, 25123, Brescia, Italy; b Department of Clinical and Experimental Sciences, SD of Infectious and Tropical Diseases, University of Brescia and ASST Spedali Civili di Brescia, 25123, Brescia, Italy

**Keywords:** Actinic keratosis, HPV, Human papillomaviruses

## Abstract

Actinic keratosis (AK) is a precancerous lesion that typically develops on photo-damaged skin, particularly in older adults and immunocompromised individuals. Due to its high prevalence and its potential to progress to cancer, AK has become an important focus of research in recent years. If left untreated, AK can evolve into squamous cell carcinoma (SCC), a type of non-melanoma skin cancer (NMSC) that carries metastatic potential.

AK is driven by multiple pathogenic mechanisms such as inflammation induced by UV radiation, oxidative stress, inhibition of apoptosis and dysregulation of the cell cycle leading to an immunosuppressive condition. In recent years, human papillomaviruses (HPVs) have also been identified as potential cofactors along with chronic sun exposure.

This article reviews the current scientific evidence on the link between HPV skin infection and AK development, with a particular focus on the potential role of HPV vaccination in managing this condition.

## 1 Introduction

Actinic keratosis (AK) is characterized by an uncontrolled proliferation of atypical epidermal keratinocytes leading to cutaneous keratinocyte dysplasia. It generally develops on sun-exposed skin with a possible evolution in the cutaneous squamous cell carcinoma (cSCC). A comprehensive meta-analysis provides an updated global prevalence rate of AK of 14 %, highlighting the significant global burden of the disease with an estimated 1.1 billion individuals affected by AK around the world [1]. This burden is expected to grow as the global population ages. However, in Europe, data about AK epidemiology are lacking and prevalence rates are extremely variable depending on study setting, characteristics and country of origin of patients [2,3]. Prevalences of 15.4 % and 5.9 % of AK have been found in UK in males and females, respectively [4]; in addition, various prevalence rates of AK have been detected, ranging from 25.3 % in Switzerland to 27.4 % in Italy, while a prevalence of 28.6 % have been observed in Spain. Then, an overall prevalence of 2.66 % for AK has been described in Germany [[5], [6], [7], [8]].

Pathogenesis of AK is complex and involves different mechanisms such as inflammation and immunosuppression induced by UV radiation, oxidative stress, modification of cell growth and differentiation pathways, tissue remodeling and inhibition of apoptosis [9]. Together with these causal factors, there is an increasing proof of evidence that the human papillomaviruses (HPVs) may play a role as cofactors in the development of AK. However, immunosuppression may favor a progression to cancer concurrently to viral infection. In fact, in patients with immunosuppression the risk of cSCC associated with the infection with HPV was elevated more than 100-fold [10,11]. On the other hand, it has been demonstrated that T-cell immunity against commensal papillomaviruses is pivotal to control skin cancer in immunocompetent individuals, and it is the loss of this immunity, rather than the effect of HPV infection, responsible of the increased risk to develop cSCC in immunosuppressed patients [12].

Therefore, in this context, the aim of this narrative review is to evaluate critically the existing evidence on the relationship between HPV skin infection and the development of AK, with a particular focus on the potential role of HPV vaccination in managing this condition.

## 2 Actinic keratosis

Skin cancer is the most frequent cancer in humans [13] and its onset is strictly related to external factors such as ultraviolet radiation exposure, the most widely recognized causal factor, even if other exposome factors seem play relevant role on the aetiology and progression of skin cancer [14]. AK, also known as solar keratosis, is one of the most frequently diagnosed skin condition by dermatologists, particularly in fair-skinned individuals with a history of prolonged sun exposure. It is considered a precursor cSCC, which represents around 25 % of all non-melanoma skin cancers (NMSC), whereas basal cell carcinoma (BCC) contributes around 75 %. Both primarily differ by its tendency to metastasize distantly. An estimated 3–7 % of patients with cSCC will develop metastasis, of whom more than 70 % will die from disease, while BCC can in rare cases metastasize and lead to death [15]. Currently, alsoHPVs as well as the skin microbiome have identified as having a role in cSCC. HPV5 and HPV8 are most detectable types in cSCC biopsies [16] and they have been classified as ‘possibly carcinogenic’ by the International Agency for Research on Cancer (IARC) [17]. An association of high risk α-HPV such as HPV16, HPV18, HPV31, HPV33, HPV45, HPV52, HPV58 with cSCC has also been demonstrated in different studies [[18], [19], [20]].Human genomic analyses have identified high-risk susceptibility genes that underlie important pathways in keratinocytes tumorigenesis as genes critical for pigment *(IRF4*, *OCA2*, *HERC2*, *TYR*, *SLC45A2, ASIP, RALY*, and *MC1R*), and HLA (*HLADQA1*) [21]. Then, other mutations have observed in key genes, such as *TP53*, *NOTCH1-2*, *FAT1*, *MLL2* [22], *CDKN2A*, *RAS*, *EGFR*, or *MYC* [[23], [24], [25]],which are associated with cancer development and progression. In AKs, besides these mutations, it has been observed the presence of epigenetic signatures; in particular, AK methylomes harbour typical features associated with the potential of developing tumour, such as CpG island hypermethylation and hypomethylation of lamina-associated domains [26]. The methylation patterns in AK tissues described by Rodriguez-Paredes et al. [26] are very similar to the cSCC methylation profile but are detected in only 50 % of AK tissues, the remaining AK samples showed patterns more closely related to healthy epidermis suggesting an origin from distinct stages of keratinocyte differentiation. Future studies focused on specific epigenetic markers may help stratify AK at higher risk of progression to SCC and what role betaHPV may have in their regulation.

AK is defined as an *in situ* carcinoma by the European guidelines [3] because it represents the initial lesion in a disease continuum and its most recent nomenclature is keratinocyte intraepidermal neoplasia (KIN). Cutaneous lesions of AK typically appear as erythematous, rough, and scaly patches, usually located on sun-exposed areas of the body such as the scalp, nose, forearms, and dorsal hands. Histologically, they present as a dysplastic proliferation of atypical keratinocytes. Since AK is considered an *in situ* squamous cell carcinoma, the dysplasia is confined to the epidermal skin layer, while the dermo-epidermal junction beneath it remains intact. AK constitutes a major public health concern and presents a significant challenge to healthcare systems, both in terms of patient care and economic costs. AK is not included in the most common cancer databases and most of its diagnoses is only based on clinical features and not on histopathological evidence [3]. A recent study [27], including patients with AK and without AK matched 1:1 on age, sex, race/ethnicity, medical centre, and date of the initial AK diagnosis and followed for 10 years, described that cSCC incidence rate was 1.92 % per year in patients with AK versus 0.83 % per year in control group. Cumulative incidence of cSCC reached 17.1 % (95 % CI, 16.9 %–17.4 %) in patients with AK and 5.7 % (95 % CI, 5.5 %-5.9 %) in control patients at the end of study. The overall AK incidence rate is approximately 1 in 15 per year, though it ranges from 1 in 60 per year in very low-risk groups to nearly 1 in 2 per year in very high-risk groups. The risk groups were evaluated by a risk prediction tool, the Skin and UV Neoplasia Transplant Risk Assessment Calculator (SUNTRAC), which on the basis of the clinical characteristics identifies the solid organ transplant recipients at higher risk for skin cancer [28].

AKs may undergo spontaneous regression, remain stable or further progress to invasive malignancy, the probability of the last type of evolution is very variable and it seems to occur in 0.1 %–16 % of pre-existing AK lesions [3] and many studies have aimed to identify the risk factors with malignant evolution of AK. The probability of an individual with AK lesions to develop cSCC is <1 % per lesion per year [29]. A study demonstrates that the presence of multiple AK lesions and their localization on the upper extremities or areas other than the head are reliable predictive factors for the risk of developing cSCC. In fact, the hazard ratio (HR) of developing keratinocyte carcinoma, comprising both squamous cell and basal cell carcinoma, is 1·68, [95 % confidence interval (CI) 1·17–2·42] for the presence of four to nine AK lesions compared with the presence of one to three AK lesions. It increases to2.·44, [95 % CI 1·65–3·61) for 10 or more lesions [30],. In general, there is a cumulative lifetime risk of 6–10 % for AK to malignant transformation into cSCC [31].

Main risk factors for AK include older age, Fitzpatrick skin type I or II and UV radiation, particularly, UV-B radiation. Although not penetrating in the skin as deep as UV-A, UV-B is more harmful as it disrupts cellular regulatory mechanisms and biological homeostasis, triggering processes of skin inflammation, oxidative stress, and DNA damage [32]. Today, UV-B is classified as a carcinogen due to its ability to mutate the TP53 gene. At the molecular level, TP53 acts as a tumour suppressor gene in the human genome, regulating essential cell cycle processes: it plays a pivotal role in controlling cell proliferation and inducing apoptosis in cells that have undergone mutations [33]. UVB exposure also prompts keratinocytes to release pro-inflammatory cytokines and stimulates the production of reactive oxygen species (ROS) in the skin, which, through oxidative mutagenesis, leads to DNA damage [34]. **Moreover, UV-B radiation has detrimental effects on the immune system, including the disruption of skin antigen-presenting cells, thereby impairing immune surveillance in the skin** [35]**. As a result of prolonged UV-B exposure, keratinocytes proliferate abnormally and uncontrollably, initially only in the basal layer of the epidermis and subsequently in the upper layers.** Therefore, the primary risk factor for the development of AK is cumulative lifelong sun exposure, which obviously correlates with age [36]. *A significant additional risk factor is male gender, which is linked to occupational sun exposure and higher rates of balding, resulting in a greater skin surface area exposed to UV-B radiation. As a result, men are affected at a higher rate of 3.5 %, compared to 1.5 % in women* [37]*. Other important risk factors include age over 45 years* [2] *and a lighter skin phototype (Fitzpatrick phototypes I and II), which are more prone to sunburn* [38]*.* The presence of multiple AK lesions within the same anatomical region often suggests a “field cancerization” effect, which is associated with a higher oncogenic risk [39]. Finally, immunosuppression, including all patients receiving immunosuppressive medication, and particularly in solid organ transplant recipients, is also strongly correlated with an increased risk of AK and these patients, often have more advanced or complex AK presentations resulting in worse treatment outcomes [28].

Diagnosis of AK)is primarily clinical and involves a thorough physical examination and dermatoscopy. According to the classification by Olsen et al., AKs are categorized into three grades based on severity: Grade 0 (barely visible or palpable lesion), Grade 1 (clearly visible and palpable lesion), and Grade 2 (prominent lesion with evident hyperkeratosis) [40]. Dermoscopy aids in identifying characteristic patterns in non-pigmented AKs, such as a red pseudo-network with fine white scales (referred to as the “strawberry pattern”), an erythematous background, and enlarged follicular openings filled with keratinous material [41]. Although clinical diagnostic accuracy is generally high (74–94 %) [42], it can be challenging due to the similarity of AK to other dermatological conditions such as seborrheic keratosis, Bowen's disease, basal cell carcinoma, and discoid lupus erythematosus. This highlights the need for additional diagnostic tools in uncertain cases [43]. In such cases, non-invasive imaging technologies, including reflectance confocal microscopy (RCM) and optical coherence tomography (OCT), are employed to obtain a more detailed analysis of the epidermal and dermal morphology [44].

The management of AK includes a broad spectrum of treatment options, which are selected based on factors such as the lesion's location, number, and the patient's overall health status [45]. Treatment approaches are generally divided into two categories: lesion-directed therapies, which target individual lesions, and field-directed therapies, aimed at treating larger areas of photodamaged skin, referred to as fields of cancerization [46].

## 3 The relationship between HPVs and AK

HPVs are a group of non-enveloped viruses belonging to the *Papillomaviridae* family with a genome composed of double-stranded circular DNA of approximately 8 kbp [47]. According to the PaVE database [48], over 200 HPV genotypes are recognized and classified into five genera (α, β, γ, Mu, and Nu). Most of them reside in the skin of immuno-competent individuals without signs of symptomatic lesions. However, the α genus includes twelve acknowledged high-risk carcinogenic HPV genotypes (HPV 16, 18, 31, 33, 35, 39, 45, 51, 52, 56, 58, and 59 included in IARC Group 1). Two of these, genotypes 16 and 18 are responsible for most HPV-related cancers including anal, cervical, oropharyngeal, penile, vaginal and vulvar cancers [49]. Other eight alpha HPVs (HPV 26, 53, 66, 67, 68, 70, 73, 82) have been classified as possibly/probably carcinogenic (IARC Groups 2A and 2B). Additionally, the α genus also includes some HPV types that can lead to benign lesions like genital or common warts (HPV 2, 3, 4, 6, 7, 10, 11, 14, 27, 28, 57) [50].

HPV genotypes of the β and γ genera are commonly found in skin epithelia [51]. It is now recognized that the β genus HPVs (including HPV types 5, 8, and 38) are involved in the early stages of non-melanoma skin cancers (NMSCs) [[52], [53], [54]]. Other HPV types belonging to the γ genus (HPV 4, 60 and 65) are also frequently detected in the skin, though no clear association with skin dysplasia or cancer have been established for these types in immunocompetent individuals but seems be associated in immunosuppressed patients [50]. Many studies suggest that the HPV, mainly from βand γ genera, may play a role in the development of AK together with chronic UV irradiation, immunosuppression and genetic features [11,55]. HPV genera potentially involved in skin carcinogenesis are reported in Table 1.

**Table 1:** Human Papillomavirus (HPV) types associated with Actinic Keratosis (AK) and/or cutaneous Squamous Cell Carcinoma (cSCC*)*.

HPV Genus	HPV Type(s)	Associated Lesion(s)	Notes	References
**α (Alpha, high-risk)**	16, 18, 31, 33, 45, 52, 58	cSCC	IARC group 1 Detected in some cSCCs	18-20, 49
**β (Beta)**	5, 8	AK, cSCC	Associated with both AK and cSCC	52-54, 56, 57, 62
	15, 20, 24, 36	AK > cSCC	Higher HPV loads in AK lesions	55, 57
	38	AK, cSCC	Enhances UV carcinogenesis	52-54, 66
**γ (Gamma)**	4	AK	High loads in AK	49, 54
	60, 65	AK	No clear association in immunocompetent individuals	50

One of the earliest lines of evidence implicating the involvement of HPVs in the pathogenesis of AK is provided by a serological study, which demonstrated the presence of antibodies against the L1 epitopes of HPV8 in large number of immunocompetent individuals with AK after adjusting for eye and hair colour and sun exposure (aOR 2.3) [56]. In a systematic review, including 2284 patients with NMSC from which 724 with AK, HPV was present in 90 AK patients (12.43 %) with 636 HPV types detected. The majority of detected HPV types belonged to β (372/635, 58.49 %) and γ (256/636, 40.25 %) genera and only a few AKs (6/636, 0.94 %) were positive for α subtypes [55]. Furthermore, Weissenborn et al. described as HPV loads (HPV5, 8, 15, 20, 24 and 36) were significantly higher in AK than in cSCC lesions [57] and more recently Galati et al. showed that gamma 1 HPV4 appeared to be strongly enriched in AK lesions compared to health skin [[49], [58]] HPV infects dividing cells of the basal layer of the epithelium and/or epidermis and then, by its proteins E6 and E7, uses cell host pioneer factors, such as KLF4, OCT4, and SOX2 toreplicate and start the process named “field cancerization” [[59], [60]]. A population-based study found that, after adjusting for sex, comorbidities, and medication confounders, a previous diagnosis of HPV infection (including both cutaneous and mucosal HPV types) increases the risk of developing skin cancer, both melanoma (aHR, 17.1; 95 % CI, 1.88–156) and non-melanoma skin cancers (aHR, 2.06; 95 % CI, 1.16–3.65) compared with the general population [61]. Another more recent serological study showed that different β HPV types and in particular HPV5 and HPV8, associated to epidermodysplasia verruciformis, are involved in the early stages of AK and in its evolution to SCC implying, therefore, that multiple HPV types might play a role in tumorigenesis [62] …

As regards the biological role of HPV4, it was shown that its E7 protein can degrade pRb and its E8 protein may interact with the calcium and integrin binding protein 1 (CIB1) [58]. This binding might interfere with the interaction of CIB1 with EVER1 and EVER 2 proteins to form a complex normally involved in the keratinocyte-intrinsic immune response to human β-papillomaviruses increasing the predisposition to be infected by them. In fact, mutations in genes codifying for EVER1 and EVER 2 proteins are a cause of epidermodysplasia verruciformis associated to higher susceptibility to symptomatic and life-long persistent β-HPV infection [63]. A recent study demonstrated in mouse models that HPV8 E6 induce keratinocyte stem cell expansion in AKs by binding p300 which phosphorylates STAT3 and activates it with a greater risk for these premalignant lesions to be more susceptible to sunlight exposition leading to their transformation in cutaneous SCC [64]. However, in healthy individuals, β-HPV may also trigger a protective immune response that prevents tumour formation [12]. The role of β-HPV in cancer development is largely dependent on the immune status of the individual, with the virus acting either as a protective factor or as a promoter of tumorigenesis [65]. Specifically, a weakened immune system leads to an increased HPV burden and greater oncogenic activity [65].

## 4 Postulated pathogenesis mechanisms

Different studies have highlighted that on the contrary of carcinogenesis induced by mucosal HPV where the oncoproteins E6 and E7 are required during all the course of neoplastic transformation, in skin carcinogenesis the process is different. Cutaneous papillomavirus proteins probably play a role only at early stages by enhancing the number of DNA mutations induced by UV radiation, and because these UV- damages are irreversible, their expression is not necessary at late stages acting therefore through a “hit and run” mechanism [65].

This may be confirmed by studies performed in transgenic mouses showing that the expression of HPV38 E6 and E7 in the skin was correlated with a higher risk of cSCC after prolonged UV exposure [53,66] This higher susceptibility to skin carcinogenesis has been associated to an accumulation of mutations, in particular in p53 and Notch genes [67]. Furthermore, a recent paper showed that the expansion of mutant p53 clones is associated to an increase of β-human papillomavirus activity and a loss of papillomavirus –specific CD8^+^ T cells [68].

This may explain why a copy of virus DNA, which is not integrated in cellular genome, is not present in all neoplastic cells. Furthermore, viral load tends to be higher in AK than in cSCC. In contrast to α-HPV associated cancers, in which HPV-DNA is detectable in tumour cells even in advanced stages, the viral DNA of βHPVs is lost in progressive stages of cSCC [57].

The principal mechanisms by which HPVs contribute to human skin cancer are described by many studies [65,69,70] and consisted in: a) deregulation of the immune system; b) inhibition of the DNAdamage response and c) inactivation of human onco-suppressors. It was demonstrated that HPVs exert their oncogenic effect through the expression of E6 and E7, which are involved in cell cycle alteration (HPV5 and HPV8 E6) and inhibition of apoptosis (HPV38E7) [62]. In particular, it was shown that E7 could down-regulate the immune response in cutaneous microenvironment helping the persistence of HPV infection and successive tumorigenesis. The postulated mechanism was the binding of HPV8 E7 with C/EBP-beta, a CCAAT-enhancer binding protein that in normal conditions determines the expression of CCL20 in keratinocytes [71]. Then, E7 of β HPV types, analogously to αHPVs, binds retinoblastoma protein (pRb) and interferes with the cell cycle determining a quiescent state during G0 and G1 phases [16]. Furthermore, it has been demonstrated that HPV5 and HPV8 E7 modifies the beta-catenin and zona occudens-1 anchor proteins, leading to an alteration of the attachment and tight junctions in epithelial cells [72]. This modification perturbs the equilibrium inside cutaneous cells and may promote epithelial-mesenchymal transition. Then, HPV8 E7 induces an increase of metalloproteases, involved in cell migration, and therefore improves the keratinocyte migration to dermis [16]. In addition, HPV8 E7 determines an increase of fibronectin and integrin alpha3 chain expression, by inducing a shift from E-cadherin to N-cadherin transcription, further contributing to epithelial-mesenchymal transition and to neoplastic transformation [73]. Regarding E6 protein, it provides the cell with an anti-apoptotic profile. In fact, opposite to what happens with alpha HPV types, E6 of different beta-HPVs does not induce the proteasomal degradation of p53 but alters its genetic expression [74].

In normal conditions, exposure to UV radiation promotes the activation of p53, which stops the cell cycle and starts the DNA repair process. However, if the exposition to sunlight is prolonged, apoptosis mechanisms are activated. In particular, the HIPK2 (homeodomain interacting protein kinase 2) has an important role in connecting the ATM (ataxia-telangiectasia mutated)/ATR (ATM- and Rad3-Related) kinases pathway to apoptosis activated by p53 [75]. Generally, after long exposition to UV radiation, HIPK2 binds p53 phosphorylating it. The phosphorylation allows p53 interaction with the CBP (c-AMP response element-binding protein) acetyltransferase, which acetylates Lys382 of p53, inducing its activation. However, HPV23 E6 can inhibit the serine phosphorylation of p53 by HIPK2 blocking the expression of pro-apoptotic genes in response to cellular damage [76] Furthermore, HPV38 E6 can block the activity of the specific histone acetyltransferase (p300), leading to inhibition of the acetylation status of p53 and consequently of its proaptoptotic function [77].

This was confirmed in K14-HPV8-E6_mutated_ and K14-HPV8-E6_wild type_ transgenic mouse models where mice carrying E6 mutant proteins lost the ability to bind p300 and therefore were not able to execute their tumorigenic activity compared to mice carrying wild type functional E proteins [78].

**Fig. 1:**
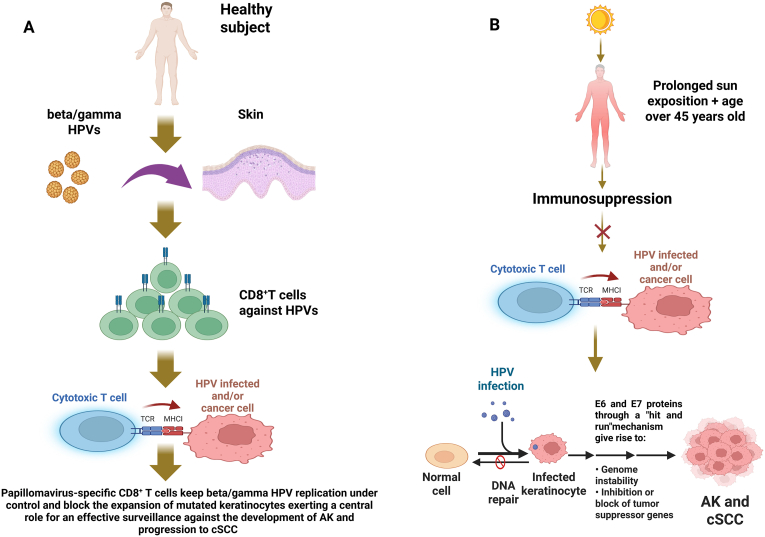
**HPVs carcinogenic activity on skin tissue.** A, effect of anti-HPV immunity on development of AK and cSCC progression; B, cofactor role of HPVs together with UV radiation in the early stages of the AK development. Abbreviations: AK, actinic keratosis; cSCC, cutaneous squamous carcinoma; UV, ultraviolet. Created by using BioRender.com.

Furthermore, E6 proteins from HPV Beta genus cutaneous papillomaviruses have been found to bind to mastermind like protein1 (MAML1), which is a coactivator for Notch-dependent transcription [79], blocking Notch signaling. In fact, MAML1 protein normally constitutes a ternary complex with CBF1(C-promoter binding factor) - ICN (intracellular domain of Notch) that is crucial for the transcriptional activation of Notch targets by engaging p300/CBP, responsible of chromatin remodeling [80] by histone acetylation. Notch generally acts as tumor suppressor; therefore, the disruption of its activity can lead to persistent proliferation of skin cells and to cancer development [62]. In addition, βHPV E6 can inhibit the DNA repair mechanism able to eliminate pyrimidine dimers produced by UV radiation by breaking down Bak (Bcl-2 homologous antagonist/killer**)** proteins, that normally induce mitochondrial pore formation and the caspase activation [81]. Furthermore,HPV38 E6 induces a higher transcription of human telomerase reverse transcriptase (TERT) mRNA able to lead to immortalisation of infected keratinocytes [82] and determines a modification of mechanisms at the base of cellular differentiation by hypermethylating the promoter of the gene that codifies for syntenin-2, a protein highly expressed in highly differentitaed keratynocytes [83]. Therefore, all these processes lead to an accumulation of mutations and modified cells, which can promote the progression to skin cancer (Fig. 1).

## 5 The role OF HPV vaccination

The observed link between HPV infection and skin lesions has driven researchers to investigate HPV-targeted strategies for both the therapeutic and preventive management of this condition. Although current HPV vaccines are designed to protect against α viral genotypes, emerging evidence points to a potential protective effect against keratinocyte carcinomas and their precursors. The potential role of HPV vaccination in the management of AK has emerged as a promising yet unexplored area of investigation. While the contribution of HPV to AK pathogenesis remains under discussion, both observational data and recent trial results indicate that HPV vaccination may benefit patients with high lesion burden and field cancerization after only two months by its administration [55,84,85].

A pivotal study in this area is the VAXAK randomized clinical trial. This double-blind, sham-controlled study enrolled 70 immunocompetent adults with multiple AKs (≥15 lesions in a 50–100 cm^2^ test area) and randomized them to receive either the 9-valent HPV vaccine or saline placebo at months 0, 2, and 6. No field-directed therapies were allowed, except for cryotherapy for thick lesions (Olsen grade II–III). At 12 months, the vaccinated group showed significantly greater reductions in lesion burden at all timepoints: 35 % vs. 25 % at month 2, 47 % vs. 29 % at month 6, and 58 % vs. 47 % at month 12. Notably, thick AKs were also significantly fewer in the vaccine group. These findings, observed in the absence of concomitant topical treatments, support the hypothesis that HPV vaccination may offer disease-modifying effects in AK through immunologic pathways [84].

This trial builds upon prior observational work by the same group. In a 2021 case series, 12 patients treated off-label with the 9-valent HPV vaccine showed an average 85 % reduction in AK lesions after one year, with visible improvement already reported at two months [86]. Another single-case report documented an immunocompetent woman with an estimated 1700 AK lesions who experienced a dramatic regression to 122 lesions within a year after receiving the HPV vaccine, without significant lifestyle changes or new therapies [87]. While these studies lack control arms and should be interpreted with caution, the consistency of clinical response across different settings is noteworthy.

Similar findings were reported by Nichols et al., who observed a reduction of 62–66 % in SCCs and complete remission of BCCs in two elderly patients with recurrent KCs following vaccination with the quadrivalent HPV vaccine [88]. Though anecdotal, these reports collectively suggest a broader anti-neoplastic potential of prophylactic HPV vaccination, possibly mediated by immune modulation.

Further indirect support comes from a retrospective study by Aoki et al., which found high-risk mucosal HPV types, particularly HPV16, in a significant proportion of cSCCs. The authors postulated that HPV vaccination could be protective because type specific, especially in younger individuals with high-risk profiles [18]. A recent systematic review synthesized molecular and epidemiological data across keratinocyte skin cancers and concluded that future vaccine strategies might benefit from targeting both alpha and beta HPV types, especially in patients with recalcitrant lesions [55]. Finally, Wang et al. highlighted a striking case of AK regression following 9-valent HPV vaccination, where lesion clearance began even before completion of the vaccination schedule. The authors emphasized the interplay between HPV oncogenes, UV-induced immunosuppression, and impaired keratinocyte apoptosis in the pathogenesis of AK, suggesting an immodulation role of mucosal HPV vaccines and laying the groundwork for immunopreventive strategies [85].

**Fig. 2:**
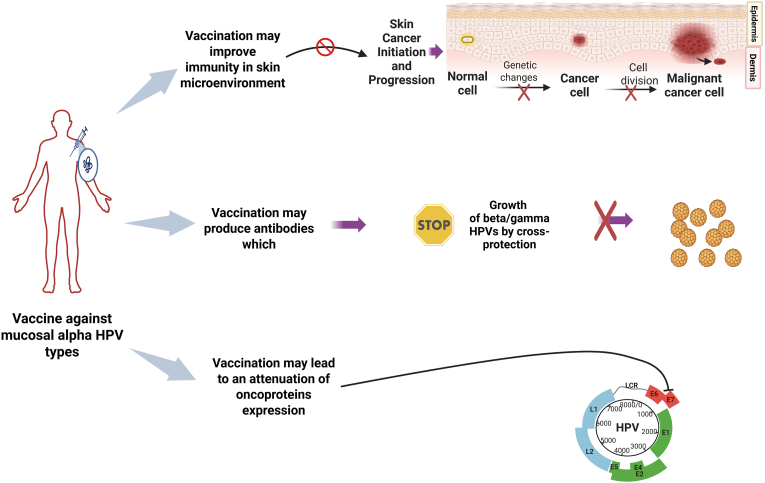
**Protective effect of anti-HPV vaccine against actinic keratosis (AK)**. Illustration of the possible mechanisms involved in the ability of vaccine against mucosal alpha HPVs to decrease the number of AK lesions and progression to cSCC. Abbreviations: cSCC, cutaneous squamous carcinoma. Created by using BioRender.com.

Although the mechanisms behind the clinical effects of HPV vaccination in AK remain unclear, several hypotheses have been proposed. One hypothesis suggests that vaccination may enhance both innate and adaptive immune responses within the skin, facilitating the local clearance of HPV-infected keratinocytes. In particular, activation of tissue-resident CD8^+^ T cells and reversal of localized immune tolerance have been discussed as possible drivers of lesion regression [86]. Another proposed mechanism involves cross-protection. Although the 9-valent HPV vaccine is designed to target mucosal α-HPV types, it may elicit immune responses that partially cross-react with β-HPV types, frequently found in AK and SCC lesions [55,88]. Beyond direct antiviral activity, HPV vaccination may also contribute to the modulation of the cutaneous microenvironment, particularly in chronically UV-damaged skin. By altering the local immunologic environment, the vaccine could help restore effective immune surveillance and reduce the permissiveness of keratinocyte precursors to neoplastic transformation [84]. A further hypothesis is that the vaccine may attenuate the expression of viral oncoproteins E6 and E7, thereby reactivating tumor suppressor pathways such as p53 and Rb. This reactivation could lead to growth arrest and apoptosis of HPV-infected, pre-neoplastic cells [85,89]. These mechanisms collectively support further investigation to clarify the role of HPV vaccination in AK management. Fig. 2 summarizes all proposed mechanisms, which can explain the role played by anti-HPV vaccination.

## 6 Future directions

The licensed HPV vaccines, which are based on L1 viral capsid protein (VLP), does not include β-HPV antigens. Therefore, the efforts have been addressed to develop also VLP- vaccines against cutaneous HPVs in order to obtain a prophylactic strategy for AK and cSCC. Because a broad spectrum of β-HPVs is frequently detected in cSCC, to achieve a universal coverage with a VLP-based vaccine constitutes a great challenge. However, different L1-based VLP vaccines against β-HPVs have been produced[[90], [91], [92]]. Despite these preclinical studies are promising, these vaccines are limited by the generation of type-specific antibodies.

Alternative approaches have been focused on producing vaccines based on antigens derived from β-HPV L2, the minor capsid protein of the virus. Because the L2 protein is well conserved between the different HPV-subtypes, this vaccine generates anti-L2 antibodies with a broader coverage by means of a cross-reactivity also against β-HPVs not included in the vaccine formulation [93]. However, the production of lower neutralizing titers led to various attempts of increasing L2 immunogenicity by linking L2 to other structures with higher immunogenicity [94,95];.

Furthermore, non-L2 chimeric VLP vaccines targeting β-HPV have been tested showing the potential of generating wide β-HPV antibody cross-reactivity [96,97].

Besides the research of vaccines committed on their ability of producing a strong antibody response, it has been taken in consideration the development of T-cell based vaccines, which can enhance cell-mediated immunity against HPV antigens. Strategies have been developed in order to induce keratinocyte to express high level of viral oncoproteins which stimulate a strong T cell immune response, able to eliminate these cells and offer a therapeutic approach to treat skin tumors [98].

Therefore, various types of vaccines against β-HPVs are under development, but further research is necessary to determine which of these vaccination strategies may better adapt to the individual patient and to the specific clinical scenario.

## 7 Conclusions

HPV types from the β, γ, μ, and ν genera, as well as certain α types, are ubiquitous on human skin and have been increasingly implicated in the development of AK and NMSC, However, up to 90 % of healthy individuals can test positive for cutaneous β-HPVs. In contrast to mucosal carcinogenesis associated with high-risk α-HPV types, in cutaneous epithelial carcinogenesis, the expression of HPV E6 and E7 oncoproteins appears to be required only at the initiation of carcinomatous transformation. These proteins facilitate the accumulation of UV-induced mutations and inhibit apoptosis in skin cells with high mutational loads through a mechanism known as ‘hit-and-run.’ This suggests a synergistic role between UV radiation and HPV in the early stages of tumor pathogenesis, though not necessarily in tumor maintenance. Currently, FDA-approved HPV vaccines targeting high-risk HPV types are based on virus-like particles composed of the major capsid protein L1. This protein is not conserved across all HPV types and exhibits varying levels of sequence homology between genera—for instance, only ∼60 % homology exists between aand β types. Consequently, the antibodies produced following vaccination are thought to be highly type-specific to α-HPVs. Nevertheless, recent findings suggest that these vaccines may have a beneficial effect on both the number and size of AK lesions, supporting the hypothesis that α-HPV vaccination could play a role in the treatment or prevention of skin cancer.

Nevertheless, the development of new vaccines that specifically target the β-HPV types mostly associated with AK and cSCC will constitute a significant step forward in prevention of skin carcinogenesis and treatment of these lesions.

## CRediT authorship contribution statement

**Maria Antonia De Francesco:** Writing – review & editing, Writing – original draft, Visualization, Validation, Supervision, Software, Methodology, Investigation, Formal analysis, Data curation, Conceptualization. **Martina Salvi:** Writing – review & editing, Writing – original draft, Visualization, Validation, Methodology, Data curation. **Roberta Gerami:** Writing – review & editing, Writing – original draft, Visualization, Validation. **Maria Alberti:** Writing – review & editing, Writing – original draft, Visualization, Validation. **Federico Cesanelli:** Writing – review & editing, Writing – original draft, Visualization, Validation. **Irene Scarvaglieri:** Writing – review & editing, Writing – original draft, Visualization, Validation. **Giorgio Tiecco:** Writing – review & editing, Visualization, Validation. **Eugenia Quiros-Roldan:** Writing – review & editing, Writing – original draft, Visualization, Validation, Supervision, Software, Methodology, Investigation, Formal analysis, Data curation, Conceptualization.

## Ethical approval

Not applicable.

## Financial support

This research received no external funding.

## Declaration of competing interest

The authors declare that they have no known competing financial interests or personal relationships that could have appeared to influence the work reported in this paper.

## Data Availability

No data was used for the research described in the article.
